# Correlation between neutrophil-to-lymphocyte ratio and postoperative mortality in elderly patients with hip fracture: a meta-analysis

**DOI:** 10.1186/s13018-021-02831-6

**Published:** 2021-11-18

**Authors:** Yu-Hang Chen, Ching-Hsin Chou, Hsin-Hsien Su, Yu-Ting Tsai, Ming-Hsiu Chiang, Yi-Jie Kuo, Yu-Pin Chen

**Affiliations:** 1 Department of General Medicine, Taipei Medical University Hospital, Taipei, Taiwan; 2 Department of General Medicine, Kaohsiung Chang Gung Memorial Hospital, Kaohsiung, Taiwan; 3 Department of Orthopedic Surgery, Wan Fang Hospital, Taipei Medical University, No. 111, Sec. 3, Xinglong Rd., Wenshan Dist., Taipei City, 116 Taiwan; 4 Department of Orthopedic Surgery, School of Medicine, College of Medicine, Taipei Medical University, Taipei, Taiwan; 5 Department of General Medicine, Taipei Tzu Chi Hospital, Buddhist Tzu Chi Medical Foundation, New Taipei City, Taiwan

**Keywords:** Hip fracture, Neutrophil-to-lymphocyte ratio, Mortality

## Abstract

**Introduction:**

The neutrophil-to-lymphocyte ratio (NLR) is a crucial prognosis predictor following several major operations. However, the association between NLR and the outcome after hip fracture surgery is unclear. In this meta-analysis, we investigated the correlation between NLR and postoperative mortality in geriatric patients following hip surgery.

**Method:**

PubMed, Embase, Cochrane library, and Google Scholar were searched for studies up to June 2021 reporting the correlation between NLR and postoperative mortality in elderly patients undergoing surgery for hip fracture. Data from studies reporting the mean of NLR and its 95% confidence interval (CI) were pooled. Both long-term (≥ 1 year) and short-term (≤ 30 days) mortality rates were included for analysis.

**Result:**

Eight retrospective studies comprising a total of 1563 patients were included. Both preoperative and postoperative NLRs (mean difference [MD]: 2.75, 95% CI: 0.23–5.27; *P* = 0.03 and MD: 2.36, 95% CI: 0.51–4.21; *P* = 0.01, respectively) were significantly higher in the long-term mortality group than in the long-term survival group. However, no significant differences in NLR were noted between the short-term mortality and survival groups (MD: − 1.02, 95% CI: − 3.98 to 1.93; *P* = 0.5).

**Conclusion:**

Higher preoperative and postoperative NLRs were correlated with a higher risk of long-term mortality following surgery for hip fracture in the geriatric population, suggesting the prognostic value of NLR for long-term survival. Further studies with well-controlled confounders are warranted to clarify the predictive value of NLR in clinical practice in geriatric patients with hip fracture.

**Supplementary Information:**

The online version contains supplementary material available at 10.1186/s13018-021-02831-6.

## Introduction

Hip fracture is a serious, devitalizing condition in the geriatric population. The global number of hip fractures is expected to exceed 6 million by 2050 [1]. Older individuals are more susceptible to hip fractures, with poor outcomes [2]. The mortality of patients with hip fracture at 30 days and 1 year after surgery is estimated to be 19% and 20–30%, respectively [3]. Hip fractures are perceived as a major threat in elderly individuals because of the high morbidity and mortality rates (14–36%) [4, 5], with physical function restored in only a few patients after surgery [6]. Hip fractures can result in an immediate public health concern due to the increasing global geriatric population. Therefore, understanding the prognosis in these individuals is highly important.

The neutrophil-to-lymphocyte ratio (NLR), a novel inflammatory marker, is the ratio of the absolute neutrophil to absolute lymphocyte count. It provides an indication of the systemic inflammatory burden [7]. NLR is relatively a cost-effective and easily accessible laboratory biomarker in routine clinical practice [8]. Several studies have indicated that NLR can predict outcomes at 30 days, 6 months, and 12 months after emergency abdominal surgery (overall accuracy for 30 days, 6 months, and 12 months was 77%, 68%, and 62%, respectively) [9] and after major vascular surgery in elderly patients [10]. Bhat et al. and Tan et al., in their reviews, concluded that NLR, as a prognostic biomarker, may be superior to white cell count or subtypes individually [11, 12].

The systemic immune-inflammation index is associated with poor all-cause mortality in older adults with hip fracture undergoing surgery [13]. Forget et al. reported that a postoperative day 5 NLR value > 5 following hip fracture surgery was associated with a high mortality risk [14]. Multiple studies have also reported that a higher preoperative NLR value is related to a higher risk of death after hip fracture surgery [15–17]. However, certain studies have found no significant association between NLR and postoperative mortality [18–20]. Clinical evidence on the association between NLR and hip fracture mortality in elderly patients remains varied, awaiting further evidence.

Considering the mixed results from current evidence, we explored the predictive value of NLR for mortality following hip fracture surgery in elderly patients. We hypothesized that a higher perioperative NLR value is associated with higher short- and long-term mortality rates after hip fracture surgery.

## Methods

### Study design and search strategy

This meta-analysis was conducted in accordance with the Preferred Reporting Items for Systematic Review and Meta-analyses guidelines [21, 22]. PubMed, Embase, Cochrane library, and Google Scholar were searched for relevant studies published from November 29, 2014 to June 30, 2021, reporting the association between NLR and postoperative mortality in elderly patients undergoing surgery for hip fracture. The search strategy included a comprehensive set of keywords: (“Neutrophil to lymphocyte ratio” or “neutrophil lymphocyte ratio” or “neutrophil-to-lymphocyte ratio” or “NL ratio” or “NLR”) and (“hip fracture” or “hip fractures”). No language restrictions were imposed and reference lists of included studies were screened.

### Eligibility criteria

Inclusion criteria were as follows: (i) studies evaluating the prognostic value of high pre- and postoperative NLR for short- or long-term postoperative mortality following hip fracture surgery; (ii) elderly patients aged ≥ 65 years; and (iii) availability of complete NLR data.

Exclusion criteria were as follows: (i) studies measuring neutrophil or lymphocyte counts separately instead of NLR, since NLR could not be calculated by the average neutrophil or lymphocyte counts in the ex post analysis (ii) studies with patients younger than 65 years; (iii) studies involving patients with multiple fractures; and (iv) studies without admission NLR data.

### Data extraction and management

Two review authors (H-HS and Y-HC) independently extracted data from individual studies. The extracted data included the study timeframe, publication year, country, design and setting, number of patients, mean age, cutoff value, pre- and postoperative NLR measurement, selection of threshold for NLR elevation, and follow-up duration. The extracted information was checked by a third author (Y-TT).

The methodological quality of each study was also assessed and scored independently by the two review authors (H-HS and Y-HC) according to Quality Assessment of Diagnostic Accuracy Studies-version 2 (QUADAS-2) [23]. In case of disagreement between the two authors, the third author (Y-TT) intervened to resolve the concern and make a final decision. The QUADAS-2 tool had four sections: patient selection, index test, reference standard, and flow and timing. The Review Manager 5.3 (Nordic Cochrane Centre, Copenhagen, Denmark) software was used to process and present the assessment results.

### Outcome measurements

The primary outcome of interest was the correlation between preoperative NLR and long-term mortality (≥ 1 year) in patients who underwent surgery for hip fracture. Secondary outcomes were the association between postoperative NLR and long-term mortality (≥ 1 year) and that between preoperative NLR and short-term (≤ 30 days) post-surgical mortality.

### Statistical analysis and data synthesis

The extracted data were combined using ReviewManager 5.3 for the meta-analysis. The differences in both pre- and postoperative NLR between survivors and nonsurvivors were estimated using mean differences (MDs) and 95% confidence intervals (CIs) from each included study. Statistical significance was defined as a *P* value of < 0.05. Heterogeneity among the included studies were assessed using the Chi-squared test (Chi2), Cochrane Q, and *I*-squared statistic test (*I*^2^). A Cochrane Q *P* value of < 0.1 and *I*^2^ values > 50% were considered significant heterogeneity [24]. A random-effects model was chosen based on the amount of heterogeneity.

In certain studies, calculations were used for some of the missing direct data. In studies that provided only the median, minimum, and maximum, we calculated the mean using Hozo’s formula [25]. In studies that did not provide the exact number of patient deaths, we multiplied the total number of patients with the mortality rate.

Possible publication biases were visually checked using a funnel plot. Egger’s statistical test was used to determine the small study effect. A statistically significant *P* value of < 0.05 suggested the presence of a small study effect.

## Results

### Identification of included studies

**Fig. 1:**
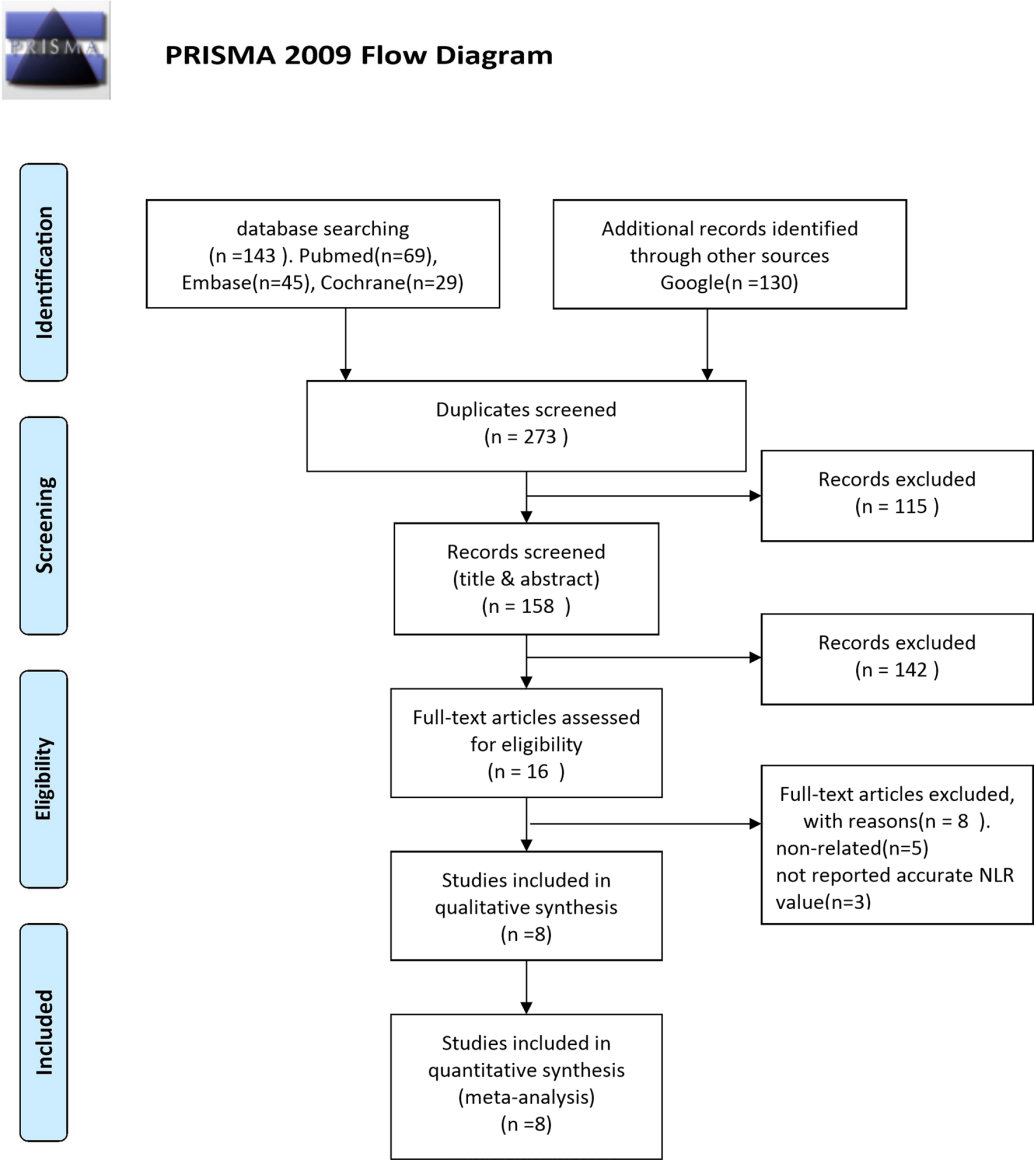
Flow diagram of study selection according to PRISMA guidelines

A comprehensive search of three databases (PubMed, Embase, and Cochrane library) led to identification of 143 relevant studies. An additional 130 studies were found through Google Scholar. These 130 studies included reports, news, and original papers. The study selection process is detailed in Fig. 1. Finally, eight studies deemed relevant were included in this meta-analysis [14–19, 26, 27]. Of these studies, five reported the predictive value of preoperative NLR on long-term mortality, four reported the predictive value of preoperative NLR on short-term mortality, and two reported the predictive value of postoperative NLR on long-term mortality.

### Quality assessment of the included studies

The methodological analysis of the included studies was based on the QUADAS-2 assessment, where the results indicated bias risk and applicability (Additional file 1: Fig. S1). In terms of patient selection and reference standards, nearly all studies demonstrated low risk, except for the study by Altinsoy et al., which showed a high risk as descriptions of the included population were lacking. In the index test, nearly all studies demonstrated an unclear risk, with a low concern of applicability. This is because the index test in these studies was a blood test for NLR and there was no description on how it was obtained. However, the blood test for NLR did not interfere with the judgement for the review question, resulting in low concerns on applicability.

### Study characteristics

The characteristics of the included studies are detailed in Table 1. All eight studies were single-center, retrospective trials comprising a total of 1563 patients. The mean age of the population was 82.26 years. Of the eight studies, four had provided their separate cutoff and respective sensitivity and specificity values. The cutoff values varied from 5.25 to 9.635 preoperatively, and only one result of postoperative cutoff value was mentioned.

**Table 1:** Characteristics of included studies

Study	Year	Country	Design, center	Models	Endpoint	Sample size	NLR cutoff	Age (years) Mean ± SD/median (min–max)	Follow-up
Bingol et al. [15]	2020	Turkey	Retrospective, single center	Preoperative NLR	30-day mortality 1-year mortality	241	6.55	81.09 ± 8.11	12 months
Özbek et al. [16]	2020	Turkey	Retrospective, single center	Preoperative NLR	1-year mortality	55	5.25	Nonsurvivors: 83.54 ± 7.3 Survivors: 79.90 ± 7.6	Maximum follow-up period of 27 months
Atlas et al. [19]	2020	Turkey	Retrospective, single center	Preoperative NLR Postoperative day 5 NLR	7-day mortality	132	Preoperative: 9.635 Postoperative: 21.07	80 ± 8.4	7 days
Temiz et al. [17]	2019	Turkey	Retrospective case control study, single center	Preoperative NLR	1-year mortality	50	4.7	Nonsurvivors: 73.81 (65–80) Survivors: 72.71 (67–80)	> 1 year
Niessen et al. [18]	2018	Belgium	Retrospective cohort study, single-center	Preoperative NLR	Hospital discharge and intra-hospital mortality	535	N/A	Survivors: 84 ± 7 Nonsurvivors: 85 ± 9	N/A
Altinsoy et al. [27]	2018	Turkey	Retrospective, single center	Preoperative NLR Postoperative day 1 NLR	Died in the ICU during the postoperative period	199	N/A	Survivors: 80.54 ± 6.942 Nonsurvivors: 85.47 ± 5.423	N/A
Sedlář et al. [26]	2015	Czech	Retrospective, single center	Preoperative NLR Postoperative NLR	5-year mortality	104	N/A	Mean age: 80 ± 9 years	48–84 (median: 60)
Forget et al. [14]	2015	Belgium	Retrospective cohort study, single center	Preoperative NLR Postoperative day 5 NLR	Patient’s survival time	242	Postoperative day 5: 5	Median age: 85 years (66–102)	21

*max* maximum, *min* minimum, *NLR* neutrophil‐to‐lymphocyte ratio, *N/A* not available, *SD* standard deviation

### Predictive value of preoperative NLR on long-term mortality

**Fig. 2:**
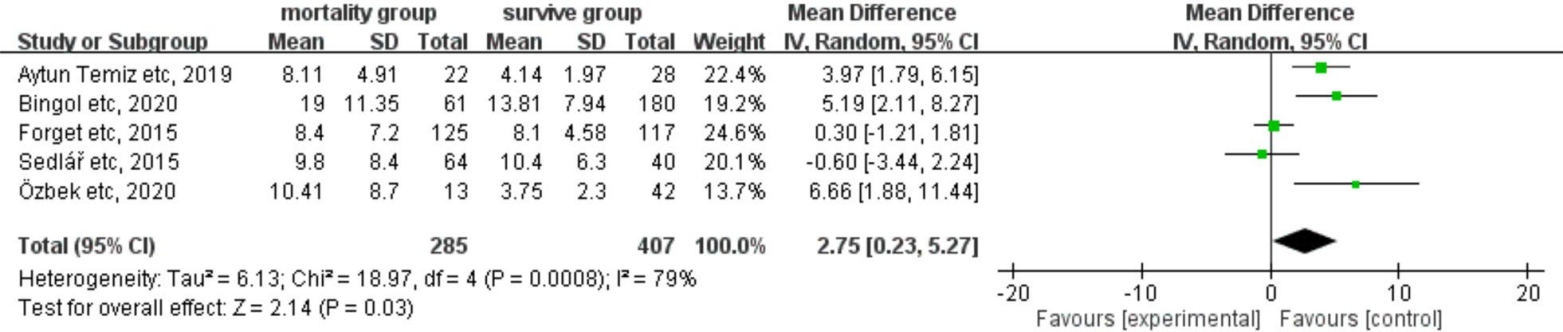
Forest plot of the mean difference in preoperative NLR between the mortality and the survival groups with a follow-up duration of > 1 year

Of the eight studies, five comprising 697 patients with hip fracture compared preoperative NLR between survivors and nonsurvivors with a follow-up duration of > 1 year (Fig. 2). Our findings revealed that preoperative NLR was significantly higher in nonsurvivors than in survivors (MDs: 2.78, 95% CI: 0.26–5.30; *P* = 0.03). However, significant heterogeneity was observed across the studies (*I*^2^: 77%, *P* < 0.05).

### Predictive value of postoperative NLR on long-term mortality

**Fig. 3:**
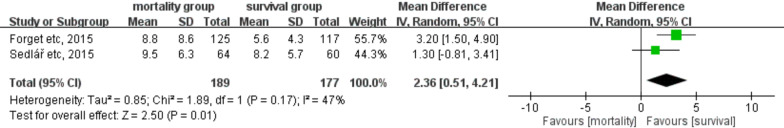
Forest plot of the mean difference in postoperative NLR between the mortality and survival groups with a follow-up duration of > 1 year

Of the eight studies, two comprising 271 patients with hip fracture compared postoperative NLR between survivors and nonsurvivors with a follow-up duration of > 1 year (Fig. 3). Our analysis revealed that postoperative NLR was significantly higher in nonsurvivors than in survivors (MDs: 2.24, 95% CI: 0.38–4.10; *P* = 0.02). In addition, no significant heterogeneity was observed across the studies (*I*^2^: 34%, *P* = 0.22).

### Predictive value of preoperative NLR on short-term mortality

**Fig. 4:**
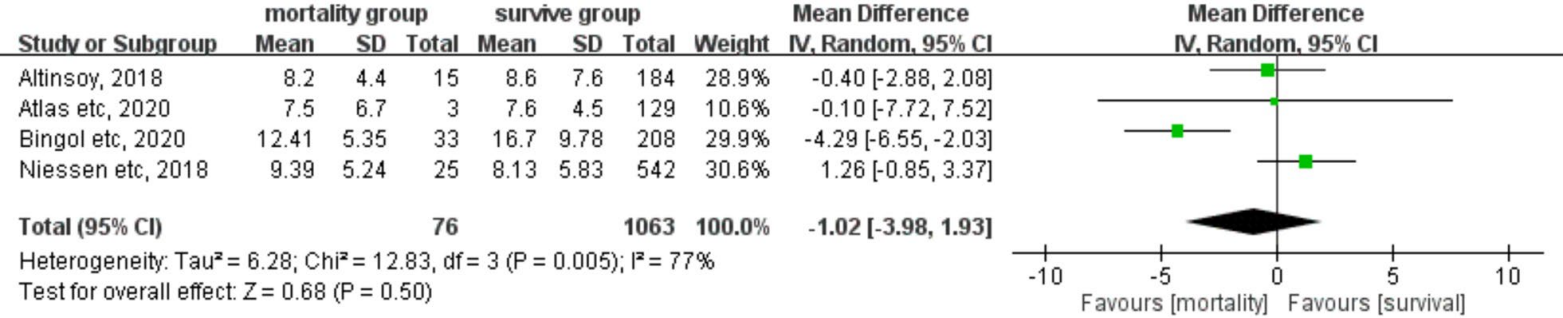
Forest plot of the mean difference in preoperative NLR between the mortality and survival groups with a follow-up duration of > 30 days

Of the eight studies, four comprising 1139 patients with hip fracture compared preoperative NLR between survivors and nonsurvivors with a follow-up time within 30 days after surgery (Fig. 4). In this meta-analysis, postoperative NLR was found to be lower in nonsurvivors than in survivors, although the difference was not significant (MDs: − 1.02, 95% CI: − 3.98 to 1.93; *P* = 0.50). Moreover, significant heterogeneity was observed across the studies (*I*^2^: 77%, *P* = 0.005).

### Publication bias

A funnel plot for the meta-analysis of the primary outcome is presented in Additional file 1: Fig. S2. Asymmetry could be visualized easily, and only five studies were included in the funnel plot. Egger’s statistical test revealed no evidence of publication bias (*t* =  − 1.417, *P* = 0.25). However, the test is more reliable when the number of studies is > 10.

## Discussion

Our meta-analysis revealed that NLR was associated with long-term mortality after hip fracture surgery. Both pre- and postoperative NLR values were significantly higher in nonsurvivors than in survivors when the follow-up duration was > 1 year. However, our results also showed that NLR failed to predict short-term mortality within 30 days after hip fracture surgery in elderly patients.

Systemic inflammation, which can be reflected by NLR, is known to be associated with prognosis following hip fracture surgery [28]. Studies have reported a similar pathophysiology of systemic inflammation and acute inflammatory markers, such as tumor necrosis factor-α (TNF-α), interleukin-6 (IL-6), and IL-10, which are associated with outcomes after hip fracture [13, 29]. In addition, elevated CRP and ferritin, which are common inflammatory markers determined in routine clinical practice, could successfully predict 30-day mortality after hip fracture [28]. However, the economic and easily accessible NLR has potential in clinical use for predicting the mortality risk following hip fracture surgery in elderly patients. This meta-analysis provides comprehensive evidence on the strong correlation between a higher NLR value and mortality risk following hip fracture surgery, offering a significant reference in clinical decision making.

In addition to indicating systemic inflammation, NLR may also reflect general health condition, such as activity of daily living, comorbidities, and nutritional status [20, 30, 31], which are crucial to the prognosis of hip fracture and its survival [32]. In a study, NLR was associated with the nutritional status. It can be a useful nutritional marker for evaluating the nutritional status of geriatric patients [33]. Inflammation was hypothesized to reduce albumin levels through reduced synthesis, increased catabolism, and translocation of albumin to extravascular pools [34]. Lower NLR level was reported to be correlated with higher albumin level and health outcomes in patients receiving hemodialysis [35]. Moreover, low serum albumin level is a sole indicator of the increased risk of in-hospital death, postoperative complications, and total mortality after hip fracture surgery in elderly patients [36]. Therefore, higher baseline NLR in geriatric patients with hip fracture may indicate poor baseline nutritional status and thereby a higher mortality risk following hip fracture surgery.

In postoperative patients with hip fracture, major cardiovascular disease is a risk factor for high mortality [20], which may be indicated by the NLR value. Systemic inflammation plays a crucial role in the pathogenesis of cardiogenic shock. In these patients, high neutrophil count was characteristic and associated with increased mortality after myocardial infarction. It was also a marker for larger area of infarction [37]. Neutrophilia may be the result of hypercholesterolemia through complicated mechanisms of enhanced granulopoiesis, mobilization from the bone marrow, and decreased clearance. The high inflammatory activity due to these interactions can lead to atherosclerotic plaques becoming unstable and prone to rupture, resulting in a cardiovascular event [38]. Peng et al. revealed that NLR is a more sensitive independent prognostic biomarker in patients with myocardial infarction than the neutrophil or lymphocyte percentage alone [39]. Patients with hip fracture are at an increased risk of both myocardial infarction and stroke up to 1 year following the hip fracture [40]. This fact can explain the finding that patients with hip fracture having higher baseline NLR may be prone to cardiovascular risk, resulting in higher mortality than patients with relatively lower baseline NLR.

In our meta-analysis, NLR was a significant predictor of long-term, rather than 30-day, mortality after hip fracture surgery in elderly individuals. The discrepancy in the weight of NLR as a predictor of short- and long-term mortality following hip fracture surgery in elderly patients may attribute to the causes of death at different postoperative time points. The most common causes of death in 30-day mortality was respiratory failure, followed by cardiac failure [41]. However, the most common cause of long-term mortality following hip fracture surgery was cardiovascular disease, followed by infectious diseases [41, 42]. Considering that NLR is a sensitive, independent prognostic biomarker in patients with myocardial infraction [39], it is sensible to associate its significance for long-term, rather than 30-day, mortality following surgery for hip fracture in this meta-analysis. On the contrary, the small number of studies included in this meta-analysis could have caused the high heterogenicity, resulting in the nonsignificance of the association between the NLR and 30-day postoperative mortality. Therefore, more evidence is warranted to clarify the discrepancy in the predictor value of NLR for the short- and long-term mortality following hip fracture surgery in elderly individuals.

NLR was associated with systemic inflammation, nutritional status, and cardiovascular risk, likely affecting the survival of elderly patients after hip fracture surgery. However, these factors were not excluded or adjusted in all the eight studies included in this meta-analysis. Thus, we could not determine which of these factors resulted in high NLR or had multifactorial associations. In a well-controlled study including patients with hip fracture, Ozbek et al. [16] found no difference in the NLR level between the deceased and survival groups. This implies the need to clarify NLR as an independent predictor of mortality risk in geriatric patients with hip fracture. To the best of our knowledge, this meta-analysis is the most extensive study investigating the association of NLR with mortality following hip fracture surgery. In addition, our study included some data points that were adjusted for potential confounding factors, making it more reliable than an individual study. With knowledge of NLR as a potential prognostic factor, clinicians can adopt a stratified care approach by prioritizing geriatric patients with hip fracture at a high risk of mortality for intensive care [43].

## Limitations

There are several limitations of this meta-analysis. First, although the association between the NLR and mortality risk after hip fracture surgery was clear, we failed to find a uniformed cutoff value with acceptable sensitivity and specificity due to lack of sufficient data. Second, mortality rates varied among studies, which may be attributed to the variations in baseline characteristics of enrolled patients in each study, resulting in heterogeneity in our reported outcomes. Age, sex, comorbidities, surgical delay, cognitive impairment, and poor renal function at presentation are important confounders and should be controlled at baseline to clarify NLR as an independent predictor of postoperative mortality in future studies. Third, all included studies were retrospective trials. Therefore, selection bias, recall bias, and other biases should be considered, which may also cause heterogeneity in pooled outcomes. Fourth, we failed to present the correlation between postoperative NLR and short term mortality (≤ 30 days) owing to the insufficient data. More studies are warranted to clarify this issue. Last, the large ethnic diversity and small sample size may cause sampling error. However, according to a statistical study, small sample size may or may not cause a noticeable bias in MDs as we expected [44]. Further studies with larger populations and a sufficient patient number are required to validate our study results.

## Conclusion

The findings of this meta-analysis revealed that higher pre- and postoperative NLR was associated with a higher long-term mortality risk following surgery for hip fracture in the geriatric population. However, whether NLR is an independent predictor of postoperative mortality risk in patients with hip fracture must be clarified by further well-controlled studies.

## Supplementary Information

**Additional file 1.** Methodological quality summary: QUADAS-2 and Funnel plot.

## Data Availability

All data generated or analyzed during this study are included in these published article [citation: 15–19, 26, 27].

## Abbreviations

NLR: Neutrophil-to-lymphocyte ratio
QUADAS-2: Quality Assessment of Diagnostic Accuracy Studies-version 2
