# Supplementary material for: Correlation between neutrophil-to-lymphocyte ratio and postoperative mortality in elderly patients with hip fracture: a meta-analysis
Source: J Orthop Surg Res. 2021 Nov 18;16:681. doi: 10.1186/s13018-021-02831-6 (PMC8600895; doi:10.1186/s13018-021-02831-6)
Supplement: Supplementary file 1 — Additional file 1. Methodological quality summary: QUADAS-2 and Funnel plot. [file 13018_2021_2831_MOESM1_ESM.docx]

**Correlation between neutrophil-to-lymphocyte ratio and postoperative mortality in elderly patients with hip fracture: A meta-analysis**


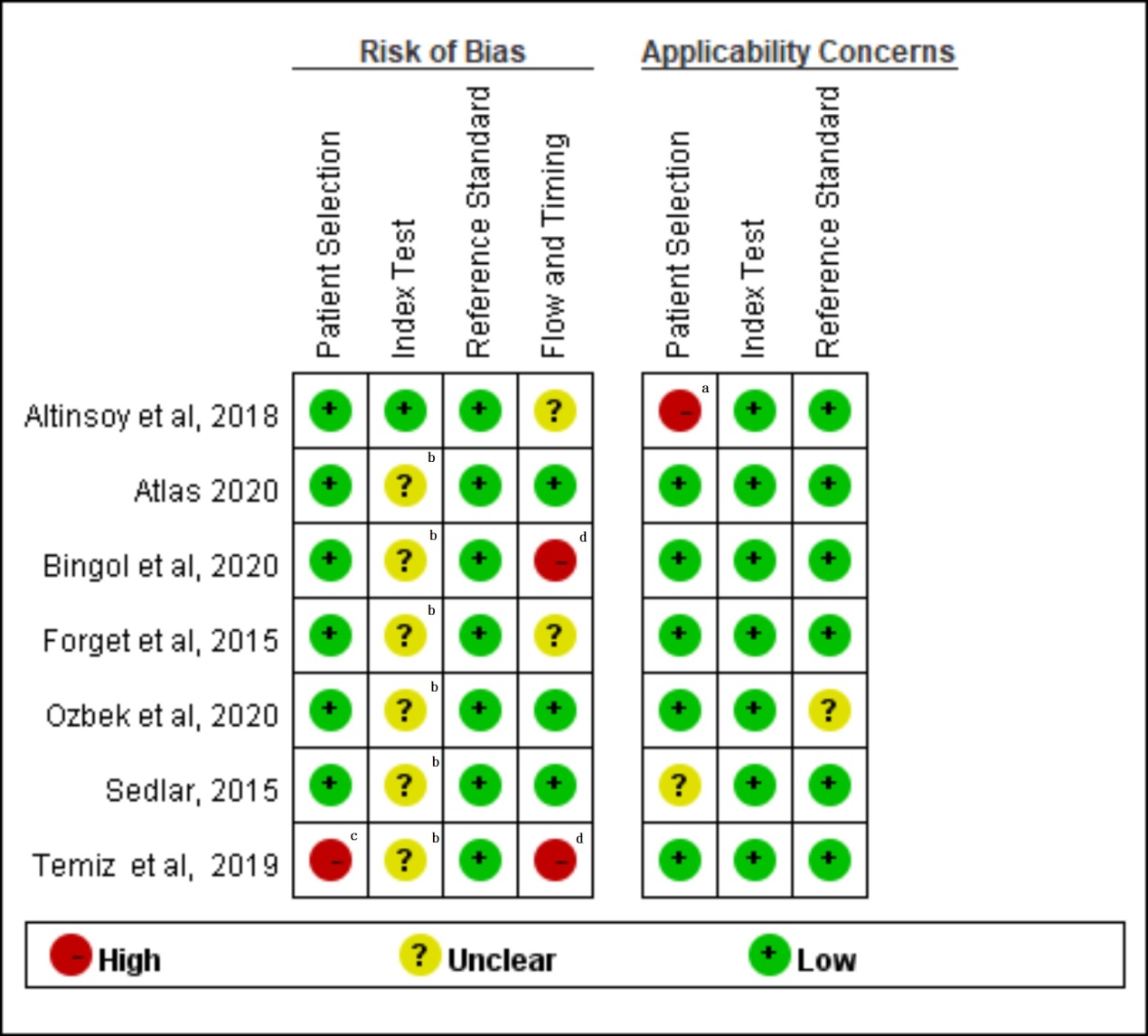

Supplementary Figure 1.

^a^Inclusion and exclusion criteria were nonspecific.

^b^The methods of how blood was collected and blood cell counts were measured could have introduced a bias.

^c^A case–control designed was not avoid and a consecutive sample of patients were not enrolled

^d^Only a small portion of the patients included in the study was included in the analysis.


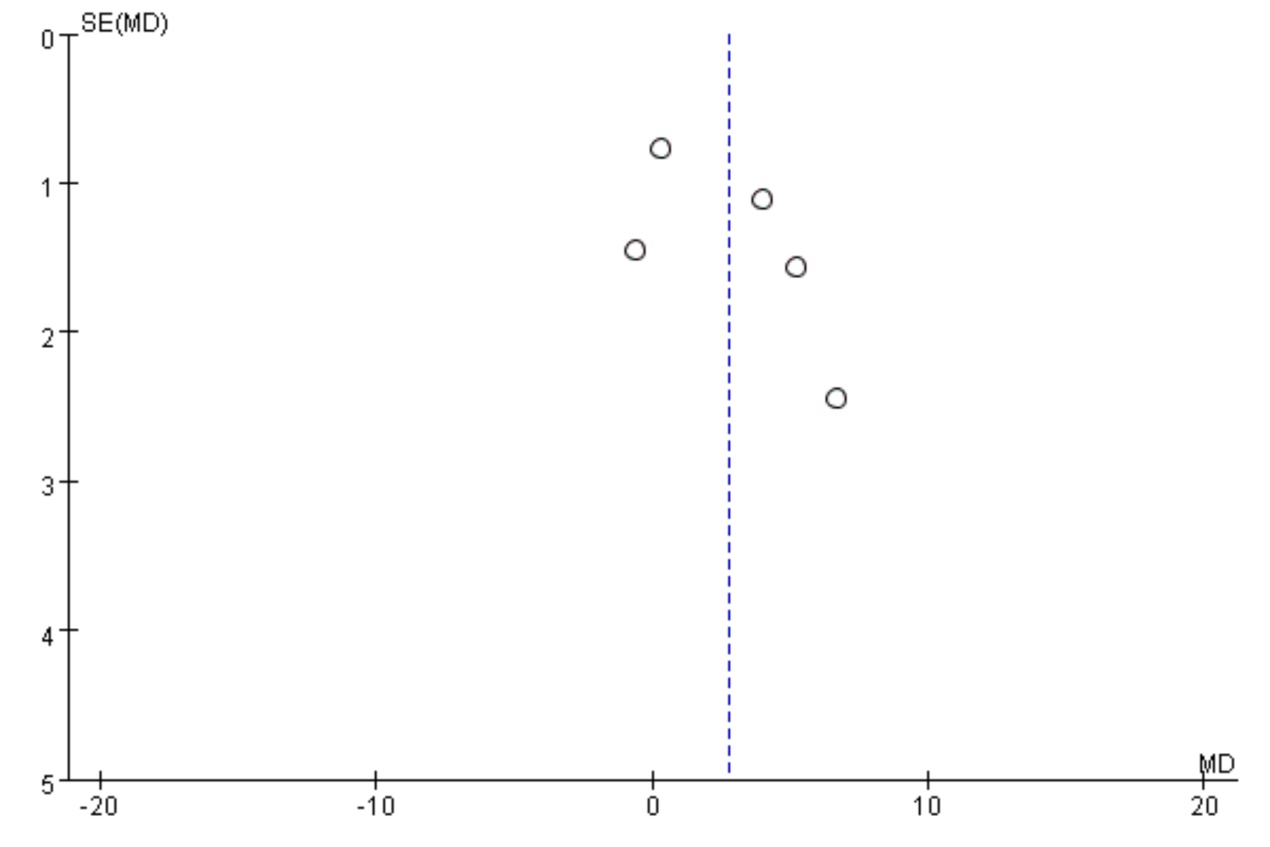


Supplementary Figure 2. Funnel plot of the studies with preoperative NLR and follow-up duration >1 year. This selection included most studies and their results demonstrate asymmetry in the graph.
